# Costs and health effects of screening and delivery of hearing aids in Tamil Nadu, India: an observational study

**DOI:** 10.1186/1471-2458-9-135

**Published:** 2009-05-12

**Authors:** Baltussen Rob, Abraham Vinod J, Priya Monica, Achamma Balraj, Anand Job, Gift Norman, Abraham Joseph

**Affiliations:** 1 Department of Public Health, Radboud University Nijmegen Medical Center, Nijmegen the Netherlands; 2 Christian Medical College, Vellore, Tamil Nadu, India; 3 Schieffelin Institute of Health – Research and Leprosy Center, Tamil Nadu, India

## Abstract

**Background:**

The burden of disease of hearing disorders among adults is high, but a significant part goes undetected. Screening programs in combination with the delivery of hearing aids can alleviate this situation, but the economic attractiveness of such programs is unknown. This study aims to evaluate the population-level costs, effects and cost-effectiveness of alternative delivering hearing aids models in Tamil Nadu, India

**Methods:**

In an observational study design, we estimated total costs and effects of two active screening programs in the community in combination with the provision of hearing aids at secondary care level, and the costs and effects of the provision of hearing aids at tertiary care level. Screening and hearing aid delivery costs were estimated on the basis of program records and an empirical assessment of health personnel time input. Household costs for seeking and undergoing hearing health care were collected with a questionnaire (see Additional file 2). Health effects were estimated on the basis of compliance with the hearing aid, and associated changes in disability, and were expressed in disability-adjusted life years (DALYs) averted.

**Results:**

Active screening and provision of hearing aids at the secondary care level costs around Rs.7,000 (US$152) per patient, whereas provision of hearing aids at the tertiary care level costs Rs 5,693 (US$122) per patient. The cost per DALY averted was around RS 42,200 (US$900) at secondary care level and Rs 33,900 (US$720) at tertiary care level. The majority of people did consult other providers before being screened in the community. Costs of food and transport ranged between Rs. 2 (US$0,04) and Rs. 39 (US$0,83).

**Conclusion:**

Active screening and provision of hearing aids at the secondary care level is slightly more costly than passive screening and fitting of hearing aids at the tertiary care level, but seems also able to reach a higher coverage of hearing aids services. Although crude estimates indicate that both passive and active screening programs can be cautiously considered as cost-effective according to international thresholds, important questions remain regarding the implementation of the latter.

## Background

The burden of hearing loss among adults is large. According to WHO estimates, some 413 million people suffer from mild or greater hearing loss globally, an estimated 187 million people from moderate or greater hearing loss, and 46 million people from severe or profound hearing loss [1]. The provision of hearing aids has been proposed as an effective approach, but a significant proportion of all people with hearing disorders goes undetected, and as a result relatively few people are fitted with hearing aids [2]. WHO estimates that only one-tenth of the global population in need of hearing aids actually receives them [2].

In this respect, two important observations can be made. Firstly, active screening for hearing disorders has been suggested as an effective strategy to increase case detection, and a number of studies in a range of developing countries have shown its feasibility in combination with provision of hearing aids [2]. However, no study has systematically evaluated the costs of such programs, let alone their cost-effectiveness in a developing country context (such studies are available on the United Kingdom [3,4], but results are obvious difficult to generalize). Second, delivery of hearing aids often takes place at tertiary health facility levels, and is managed by specialized audiologists. There is little structured knowledge as to whether hearing aids can also be effectively delivered at lower, e.g., secondary care level, and on the costs (or savings) associated with these alternative strategies. There is an urgent need for research to answer these questions [2].

In response to these two observations, this paper reports on the costs and health effects of two active screening programs in the community in combination with the provision of hearing aids at secondary care level, and the costs and effects of the provision of hearing aids at tertiary care level in three sites in Tamil Nadu, India, in the period 2007–2008. This study is part of a number of WWHearing pilot projects on the delivery of hearing aids in developing countries [5].

## Methods

The study evaluated three different programs:

1. Active screening and fitting at secondary care level

Active screening in 62 communities in Karigiri through hearing camps and fitting of hearing aids to 101 patients by the community hearing workers in the Karigiri secondary level health facility. This program also fitted hearing aids to 111 patients who presented themselves directly at Karigiri secondary level health facility for treatment;

2. Active screening and fitting at secondary care level

Active screening in 63 communities in Vellore through hearing camps and fitting of hearing aids to 163 patients by the community hearing workers in Community Health and Development (CHAD), a department of the Christian Medical College, Vellore providing primary and secondary health care. This program also fitted hearing aids to 32 patients who presented themselves directly at CHAD for treatment;

3. Passive screening and fitting at tertiary care level

Passive screening and fitting of hearing aids to 100 patients in the ENT department of the Christian Medical College (CMC), a tertiary level health facility in Vellore.

In both active screening models (programs 1 and 2), community hearing workers screened adults in the community. A selection of those receive a hearing assessment, for eligible patients an ear mould impression was taken, and the impressions were then sent for conversion into ear moulds at tertiary care level (CMC). Subsequently, at the secondary level health facility, the programmable hearing aid was fitted by the community hearing worker. In both programs 1 and 2, all people received monaural hearing aids. People were referred in cases where disorders were not treatable with hearing aids. The patient was followed-up in a total of four visits within a year (at 2 weeks, 1 month, 3 months and 6 months) and after that at yearly intervals, also by the community hearing worker. The programs also served patients who present themselves directly for treatment. Community hearing workers attended a three week training program on basic hearing health care, including basic audiometry, ear mould impression taking and hearing aid fitting.

In the passive screening mode (program 3), patients presented themselves at the outpatient department of the tertiary level health facility, and were referred to the audiology department in case of hearing problems. The consultation with the audiologist included a physical and a standard audiometric examination. People were referred in cases where disorders were not treatable with hearing aids. For patients eligible for a hearing aid, an ear mould impression was taken by an ear mould technician. At a second visit, a digital, programmable hearing aid was fitted. In program 3, some people received monaural and some binaural hearing aids. The patient was followed-up in a total of four visits within a year (at 2 weeks, 1 month, 3 months and 6 months) and after that at yearly intervals.

In all programs, eligibility of patients for hearing aids was determined with reference to WHO Guidelines for Hearing Aids and Services for Developing Countries [2]. In all programs, based on the diagnostic audiological assessment, hearing aids were programmed to NAL-NL1 prescription standards [4] and fitted by the audiologists and hearing care workers involved in the project. Evaluation of each individual fitting was later performed and this included aided hearing threshold measures, and speech detection testing. Results from these measures will be considered in a future research report. The hearing health care service was provided free of health care costs to the patients.

The study had an observational design, in which all people were followed during the course of their inclusion in the program. The costing analysis followed WHO guidelines on costing and cost-effectiveness analysis [7] where possible, and was based on the ingredient approach, i.e., separate reporting of prices and quantities. All costs were estimated with base year 2007. Health care costs included those of screening and hearing aid delivery, and were estimated on the basis of a detailed assessment of program records and an empirical assessment of health personnel time input. The latter was obtained through a simple registration of the time that the health personnel (audiologists, hearing worker, or ear mould technician, depending on the study site) spent on care for each person. These data were collected for all programs. Household costs for seeking and undergoing care were collected with a questionnaire, administered by the health personnel to the patients. This included questions not only on costs of travel and lodging, but also on foregone income, i.e., time lost because of the program. In addition, household treatment seeking patterns prior to the present programs were assessed, including questions on associated costs. These data were collected for all programs.

Guidelines on cost-effectiveness analysis (CEA) typically advocate adoption of the societal perspective in the calculation of costs and effects [7,8]. This refers to the inclusion of all changes in resource use, no matter who is paying the costs, and includes health care costs (i.e., those that accrue to the health care sector) and household costs (i.e., those that accrue to patients and families). However, in practice, cost-effectiveness analysis often only includes health care costs, and one of the reasons for this is the difficulty of valuing and, therefore, estimating time costs [8]. As a compromise, we separately report the household costs of seeking and undergoing care in the context of the present program. In addition, we separately report household costs of seeking and undergoing care *prior *to enrolment in the present program.

We only include health effects and not effects of improved hearing on labor productivity. To our knowledge, no study has assessed the health state valuation of deafness for adults in India, and following WHO guidelines on CEA, we use health state valuations from the Global Burden of Disease study [9]. The health state valuation for deafness in adults (15 years and older) equals 0.216 (untreated) and 0.168 (treated). Subsequently, disability adjusted life years (DALYs) averted were calculated for the people included in the study. Since follow-up of the fitted person was only limited in the study, we assumed a use of the hearing aids for five years. In other words, every person fitted with hearing aids gains 0.168 DALYs [calculated as: 5*(0.216–0.168)], and discounted by 3% following WHO guidelines on CEA [7]. We did not include any health effects of the programs after this five year period.

The study was performed in the context of the routine service program, and no specific ethical approval was required. All respondents gave informed consent to their participation in this study.

## Results

Patient volumes and costs for the various programs are summarized in Table 1 (details are available in Additional file 1). The total number of people screened at camps equaled 1,926 in program 1 and 1,648 in program 2. A proportion of these were referred to the respective health facilities, and 101 of these were fitted with hearing aids at the secondary care level in program 1 and 163 in program 2. In addition to these people identified through active screening, other people presented themselves directly for treatment at the health facilities. Of those, 111 were fitted with hearing aids at the secondary care level in program 1, and 32 in program 2. At the tertiary care level, 100 people were fitted with hearing aids (50 monaural and 50 binaural).

**Table 1:** Costs, effects and cost-effectiveness of different interventions

	Program 1	Program 2	Program 3
***Costs***			
Number of people			
People screened at camps	1,926	1,648	
Identified at camps	588	534	
Fitted with hearing aids, identified through camp (a)	101	163	
Fitted with hearing aids, presented at clinic (b)	111	32	100
Total number of patients fitted with hearing aids © = (a)+(b)	212	195	100
			
Total costs			
Fixed project costs (d)	352,631	389,601	
Organisation of camps (e)	97,224	64,340	
Fitting and follow up, identified through camp (f)	440,975	681,595	
Fitting and follow up, presented at clinic (g)	613,961	247,584	569,332
Total (h) = (d)+(e)+(f)+(g)	1,504,790	1,383,120	569,332
Total (US$) (i)	32,154	29,554	12,165
			
Average cost per patient			
Fixed project costs (j) = (d)/(c)	1,663	1,998	-
Organisation of camp (k) = (e)/©	459	330	-
Fitting and follow up (l) = ((f)+(g))/(c)	4,976	4,765	5,693
Total (Rs) (m) = (j)+(k)+(l)	7,098	7,093	5,693
Total (US$) (n)	152	152	122
			
***Health effects***			
DALYs per patient fitted (p)	0.168	0.168	0.168
Total DALYs averted (q) = (c)*(p)	36	33	17
			
***Cost-effectiveness***			
Cost (Rs) per DALY averted (r) = (m)/(q)	42,250	42,220	33,889
Cost (US$) per DALY averted (s) = (n)/(q)	903	902	724

Health care costs of the active screening programs included fixed and variable costs (Additional file 1). Fixed costs are defined here as costs that do not vary with the number of people screened, and include costs of personnel involved in the program (such as that of management, and of community hearing workers), equipment (such as portable audiometers and otoscopes purchased by the program), and materials. These costs are almost equal in the two sites where the screening programs took place. Variable costs are defined here as costs that do vary with the number of people screened (and thus with the number of camps) and/or treated. In program 1, 62 camps were organized at a total costs of Rs. 97,224 (US$2,077), whereas in program 2, 63 camps were organized at a total cost of Rs. 64,340 (US$1,375). Other variable costs include that of mould impression materials, hearing aids, and outpatient visits for consultation and follow-up of patients in the health facilities (either referred through camps, or presented directly at the health facilities).

Total costs of the programs ranged from Rs 569,332 (US$12,165) in program 3 to Rs 1,383,120 (US$15,280) in program 2. The total costs per person fitted with hearing aids ranged from Rs 5,381 (US$122) in program 3 to around Rs. 7,000 (US$152) in programs 1 and 2 (Table 1). The health effects per person fitted with hearing aid equals 0.168, and multiplied by the number of hearings fitted in the various sites, the number of DALYs averted ranged between 17 (program 3), 33 (program 2), and 36 (program 1). The cost per DALY averted ranged from around Rs 34,000 (US$720) in program 3, to Rs. 42,000 (US$900) in programs 1 and 2 (Table 1).

Additional file 1, Table S4 summarizes the main observations from the survey on patient treatment patterns and costs. In all sites, there is a diversity of main income-generating activities. On average, people suffered from hearing impairment between 116 and 139 months, i.e. around 10 years, before enrollment in the present program. The majority of people being fitted at the primary and secondary care levels did consult another provider for treatment of hearing disorders before enrolment in the present program, whereas for the vast majority of people fitted at tertiary care level, this was their first provider. In case other providers were consulted, this was a private provider in the vast majority of cases. In all sites, few people had consulted a second other provider. People paid expenses for fees, drug and tests, transport costs and faced income loss when consulting providers prior to the present program.

Of patients included in programs 1 and 2, the majority was identified in the camps through active screening. In program 3, all patients presented themselves at the health facility. Patient costs in terms of fees, drugs, tests related to the first contact in the present program were nearly absent at the secondary care levels, and significant at tertiary care level. Income loss of patients and accompanists because of seeking and undergoing care ranged between Rs.12 (US$0,26) and Rs. 53 (US$1,13) between the different visits and sites. Costs of food and transport ranged between Rs. 2 (US$0,04) and Rs. 39 (US$0,83).

Sensitivity analysis was applied to test the robustness of results towards the use of alternative values on key parameters, including the proportion of patients actually wearing hearing aids, the lifetime of the hearing aids, the difference in health state valuation between treated and untreated deafness, and cost of hearing aids. This has a varying impact on study results, individually, and in combination (in a so-called worst case analysis) (Table 2). Also, we observed that the proportion of people directly presenting to the clinic was much higher in program 1 compared to program 2 (109% of those identified through camps in program 1, versus 20% of those identified through camps in program 2). If we assume 109% of those identified in the community in program 2 to present themselves directly in the clinic, cost per DALY decreased to Rs. 32,084 (US$685).

**Table 2:** Sensitivity analysis

Scenario (number, variable)		Base-line value	Sensitivity analysis	Cost per DALY averted		
				Program 1	Program 2	Program 3
0	Baseline			903	902	724
1	Proportion of patients wearing hearing aid	70%	50%	1264	1,263	1,014
2	idem	70%	90%	702	702	563
3	Lifetime hearing aid (years)	5	3	1,505	1,504	1,207
4	idem	5	7	645	644	517
5	Difference in health state valuation treated and untreated deafness	0,048	0,024	1,806	1,804	1,448
6	idem	0,048	0,072	602	601	483
7	Fitted with hearing aids, presented at clinic (program 2 only)	32	179	NA	685	NA
8	Cost of hearing aid	Rs. 1,680 (US$36)	Rs. 3,000 (US$64)	1,071	1,070	976
9	idem	Rs. 1,680 (US$36)	Rs. 1,000 (US$21)	816	816	594
10	Worst case scenario (scenarios 1, 3, 5, and 8 combined)	Combined	Combined	4,996	4,993	4,554

## Discussion

Active screening for hearing impairment, including the provision of hearing aids to eligible patients at primary or secondary care level, is slightly more costly than fitting of hearing aids to those people who present themselves for treatment at the tertiary care level. Cost differences between the different programs – as cost per patient fitted with hearing aids – are maximally some 25%, and can be, given uncertainty about study assumptions, considered to be moderate only. Active screening programs seem to be better able than passive screening programs to reach a higher coverage of hearing aids services in the community, and whether this is worth the slightly higher costs is a matter of judgment.

A number of issues need to be taken into account when considering the costs estimates. First, and most obvious, active screening programs do require significant human and material resources, and these costs add to the average costs of hearing aids fitted. The cost savings stemming from treatment at secondary care level instead of treatment at tertiary care level, do offset these costs to a certain extent. Second, programs 1 and 2 basically concern two components, i.e. an active screening component in the community that refers people to the respective health facility, and another component that involves people who present themselves directly at the health facility for treatment. We analyze the components in combination, as the second component would not exist without the first component being in place (the information campaign in the active screening program has likely prompted some people to directly present themselves at the health facility for treatment of hearing disorders, whereas prior to the program, no specific hearing treatment was available).

In addition to the detailed cost estimates, this paper also made some crude estimates of related health effects, and cost-effectiveness of the hearing aid delivery models. We estimated that the cost per DALY averted ranged between around US$720 (passive screening) and US$900 (active screening). WHO labels interventions as cost-effective if they cost less than three times GDP per capita, and as not cost-effective if they cost more than three times GDP per capita [10]. India has a gross domestic product (GPD) per capita of US$871 per 2007 [11]. This indicates that, on the basis of our base-line estimates, both the passive and active screening programs can be considered as cost-effective interventions according to international thresholds. However, sensitivity analysis reveals that alternative values of key variables can have an important impact of study results, and e.g. can almost double the cost per DALY averted in case assumptions on the life time of hearing aids, or the gain in health state valuation are changed. In a worst case scenario, the cost per DALY may increase more than five-fold. Consequently, the above findings and statement on economic attractiveness needs to be interpreted with caution.

An interesting observation is that the majority of patients in the active screening programs had visited a different health provider before being included in the program under study. This indicates that the hearing disorders of the people in the present program did not go undetected prior to the active screening program, but rather that people did not seek care with appropriate health providers. One possible explanation is that hearing aids were not delivered free of charge prior to the present program (as they were in the present program), but at significant costs (similar or above the price as used in our analysis of Rs. 1680 (US$36)), and may not have been affordable to households. This poses a question concerning the relevance of the screening program, and whether the delivery of (highly) subsidized hearing aids, in combination with an (community-based) information campaign publicizing their availability, may not reach the same objective.

Our study has a number of limitations. Firstly, we only made a crude assessment of the health effects of hearing aids by using health state valuations derived from the Global Burden of Disease study. This approach does not differentiate between the capacities of the secondary and tertiary health facility levels to diagnose hearing problems and fit hearing aids. However, recent analyses shows that the secondary and tertiary care health facilities as included in our study, are equally effective in that respect [12]. Second, our health effects estimates did take into account compliance of 70% in terms of wearing hearing aids. Lack of compliance in use is a substantial problem everywhere among elderly and child hearing aid users, including those in developing countries [13], and it is not sure whether our assumptions is realistic. Sensitivity analysis shows a modest impact on study results. Third, the analysis only included the effects of hearing aids on health, and not on productivity losses. Several studies have indicated that such losses may be considerable [14,15]. If these were included the programs would be rendered more cost-effective.

Fourth, if screened people are referred for consultation, but are not found eligible for hearing aids, health facilities provide additional diagnostic tests for and treatment of ear-related health problems as a standard procedure (as such, the screening program also contributes to towards an integrated primary health care approach). Related health effects and costs were not included in the analysis, as the decision to carry out these diagnostic tests and treatment stands by itself [7] and should be subjected to a separate economic analysis. However, related costs may be significant and equally relevant in similar contexts, and policy makers should take these into account when making decisions about the funding of the programs at hand. Fifth, programs were evaluated in two different districts (program 1 in Karigiri, and programs 2 and 3 in Vellore). Differences in the epidemiology of disease, and health care organization between the districts may have confounded the results, as well as differences in the organization of the screening programs. For example, the effectiveness of screening appeared higher in program 2 compared to program 1 (163 identified out of 1,648 screened people versus 101 identified out of 1,926 screened people), while fewer people in program 2 presented directly at the clinic as compared to program 1 (32 versus 111). The reason for these differences is not clear. However, sensitivity analysis shows only a marginal effects on cost-effectiveness results, and our study conclusions therefore remain unchanged. Sixth, screening activities of community workers may not necessarily be limited only to hearing impairment, but may also include other diseases and impairments, which would decrease the costs of hearing impairment screening programs as well as promote integration of services. Seventh, this study has evaluated a universal screening program, and it is not clear how its results compare to selective screening of high risk populations, and whether this would be more economically attractive, and therefore a preferred program.

Baltussen et al. [16] recently reported on costs of three school-based screening programs in combination with the provision of hearing aids at respectively primary, secondary and tertiary care level in China. The study concluded that, in combination with screening, the provision of hearing aids is least costly at primary care level (US$227 per child fitted), followed by secondary care level (US$277) and tertiary care level (US$365). Indian and Chinese programs cannot be fully compared as the tertiary care level program in China includes a screening component, whereas the tertiary care level program in India study does not. Nevertheless, this leads to a number of observations. Firstly, the costs per patient fitted with hearing aids is lower in the community screening program in India than in the school-based screening program in China. The cost differences may be explained by many factors, including differences in the epidemiological profile of hearing impairments between adults and children, and between India and China, differences in countries' price levels, and differences in the nature of the screening programs themselves. It is thus difficult to draw any conclusion on the relative economic attractiveness of school-based versus community-based screening for hearing impairment. Secondly, as in the present Indian study, the Chinese study reveals only relative small cost differences between the different programs.

Based on the present study and the study in China [16], a number of research recommendations can be made. Firstly, better information is required on the compliance of people with hearing aids, and on the lifetime of hearing aids in countries as India and China. Also, the health state valuations of treated and untreated hearing impairment are largely Second, local studies can be useful to inform local policy making, but are difficult to generalize and therefore hold little relevance for other policy making context. The above discussion on the comparison between Indian and Chinese study results illustrate this. We therefore argue for multi-country studies that evaluate similar interventions, and that allow the study of (common) contextual factors, such as epidemiology of disease, health care seeking behavior, the structure of the health care system, and price levels, impacting on costs and health effects. It is only by the identification of those factors that results of economic analysis hold broad relevance, i.e. also beyond the countries directly involved in a (multi-country) study.

## Conclusion

Active screening and provision of hearing aids at the secondary care level is slightly more costly than passive screening and fitting of hearing aids at the tertiary care level. However, the former carries the benefit that it seems able to reach a higher coverage of hearing aids services and it can be combined with the screening and management of other diseases in the community. Although crude estimates indicate that both passive and active screening programs can be cautiously considered as cost-effective according to international thresholds, important questions remain regarding the implementation of the latter.

## Competing interests

The authors declare that they have no competing interests.

## Authors' contributions

RB, VJA, JA & NG designed the study. VJA, NG, BA, JA, JA, and MP collected the data. RB analyzed the data. All authors contributed to the drafting of the manuscript.

## Pre-publication history

The pre-publication history for this paper can be accessed here:



## Supplementary Material

Additional file 1 **Appendix tables**. Tables forming appendix Click here for file

Additional file 2 **Appendix questionnaire**. Questionnaire about the costs of hearing problems and its treatment. Click here for file
