# Supplementary material for: Costs and health effects of screening and delivery of hearing aids in Tamil Nadu, India: an observational study
Source: BMC Public Health. 2009 May 12;9:135. doi: 10.1186/1471-2458-9-135 (PMC2695455; doi:10.1186/1471-2458-9-135)
Supplement: Additional file 1 — Appendix tables. Tables forming appendix [file 1471-2458-9-135-S1.doc]

| Appendix Table 1. Patient volumes and resource use in program 1 | |  |  |  |  |
| --- | --- | --- | --- | --- | --- |
| **Patient numbers** | | Katpadi | Gudiyatham | total |  |
| At camps | |  |  |  |  |
|  | People screened | 948 | 978 | 1.926 |  |
|  | Consultations at 2nd level hospital, referred through camps | 276 | 312 | 588 |  |
|  | Impressions taken | 67 | 40 | 107 |  |
|  | Fitted with hearing aid | 66 | 35 | 101 |  |
| Directly at clinic | |  |  |  |  |
|  | Patients consulted | 347 | 452 | 799 |  |
|  | Impressions taken | 68 | 62 | 130 |  |
|  | Hearing Aid Fitted | 55 | 56 | 111 |  |
|  |  |  |  |  |  |
| **A. Fixed project costs** | | Units | Unit price | Costs | **Total costs** |
| *Training costs* | |  |  |  |  |
|  | Per diem for CHW trained (during 3 months, 3300 per month) | 3 | 9.900 | 29.700 |  |
|  | Annualized cost (useful life 5 years) |  |  | 6.485 |  |
| *Personnel* | |  |  |  |  |
|  | Project coordinator (5% FTE) | 1 | 336.000 | 16.800 |  |
|  | Community Hearing Workers (full-time) | 3 | 90.779 | 272.337 |  |
|  | Coordinator Community-based rehabilitation workers (20% FTE) | 1 | 72.000 | 14.400 |  |
|  | Community-based rehabilitation (CBR) workers | 14 | 1.800 | 25.200 |  |
|  | Total costs |  |  | 328.737 |  |
| *Equipment (unit costs are annualized costs)* | |  |  |  |  |
|  | Otoscope | 3 | 293 | 880 |  |
|  | Audiometer | 3 | 5.459 | 16.377 |  |
|  | Ice box | 1 | 152 | 152 |  |
|  | Total costs |  |  | 17.409 |  |
| Total costs | |  |  |  | **352.631** |
|  |  |  |  |  |  |
| **B. Screening in community** | |  |  |  |  |
|  | Number of camps | 62 |  |  |  |
|  | Cost per camp (materials, vehicles etc excl. salaries) | 1 | 807 | 807 |  |
|  | Driver (hours per camp) | 8 | 76.800 | 320 |  |
|  | Community health volonteers (hours per camp) | 16 | 2.880 | 24 |  |
|  | CHP (hours per camp) | 17 | 13.200 | 117 |  |
|  | Camp organisers (hours per camp) | 8 | 72.000 | 300 |  |
|  | Total costs |  | 1.568 | 97.224 |  |
| Total costs | |  |  |  | **97.224** |
|  |  |  |  |  |  |
| **C. Follow up of screened patients at primary care level** | | unit (minutes) | unit cost (15 min) | costs |  |
| Visit 1 (consultation) | |  |  |  |  |
|  | Number of patients | 588 |  |  |  |
|  | Costs of outpatient visits | 33 | 82 | 181 |  |
|  | Total costs | 588 | 181 | 106.536 |  |
| Visit 1 (impression making) | |  |  |  |  |
|  | Number of patients | 107 |  |  |  |
|  | Mould impression materials | 1 | 25 | 25 |  |
|  | Total costs | 107 | 25 | 2.675 |  |
| Other visits | |  |  |  |  |
|  | Number of patients | 101 |  |  |  |
|  | Visit 2 (fitting) | 30 | 82 | 165 |  |
|  | Visit 3 (follow up) | 15 | 82 |  |  |
|  | Visit 4 (follow up) | 15 | 82 |  |  |
|  | Visit 5 (follow up) | 15 | 82 |  |  |
|  | Visit 6 (follow up) | 15 | 82 |  |  |
|  | Visit 7 (follow up) | 15 | 82 |  |  |
|  | Total costs | 101 | 165 | 16.636 |  |
| *Equipment costs* | |  |  |  |  |
|  | Hearing aids | 101 | 1.727 | 174.455 |  |
| Total costs | |  |  |  | **300.302** |
|  |  |  |  |  |  |
| **D. Assessment and follow up of patients presented directly at primary care level** | | | |  |  |
| *Number of patients* | | 799 |  |  |  |
| *Costs of outpatient visits* | | unit (minutes) | unit cost (15 min) | costs |  |
| Visit 1 (consultation) | |  |  |  |  |
|  | Number of patients | 799 |  |  |  |
|  | Costs of outpatient visits | 30 | 82 | 165 |  |
|  | Total costs | 799 | 165 | 131.605 |  |
| Visit 1 (impression making) | |  |  |  |  |
|  | Number of patients | 130 |  |  |  |
|  | Mould impression materials | 1 | 25 | 25 |  |
|  | Total costs | 130 | 25 | 3.250 |  |
| Other visits | |  |  |  |  |
|  | Visit 2 (fitting) | 30 | 82 | 165 |  |
|  | Visit 3 (follow up) | 15 | 82 | 82 |  |
|  | Visit 4 (follow up) | 15 | 82 | 82 |  |
|  | Visit 5 (follow up) | 15 | 82 | 82 |  |
|  | Visit 6 (follow up) | 15 | 82 | 82 |  |
|  | Visit 7 (follow up) | 15 | 82 | 82 |  |
|  | Total costs | 111 | 576 | 63.991 |  |
| *Equipment costs* | |  |  |  |  |
|  | Hearing aids | 111 | 1.727 | 191.728 |  |
| Total costs | |  |  |  | **390.574** |

| **C. Follow up of screened patients at secondary care level** | | unit (minutes) | unit cost (15 min) | costs |  |
| --- | --- | --- | --- | --- | --- |
| Visit 1 (consultation) | |  |  |  |  |
|  | Number of patients | 588 |  |  |  |
|  | Costs of outpatient visits | 33 | 176 | 388 |  |
|  | Total costs | 588 | 388 | 228.209 |  |
| Visit 1 (impression making) | |  |  |  |  |
|  | Number of patients | 107 |  |  |  |
|  | Mould impression materials | 1 | 25 | 25 |  |
|  | Total costs | 107 | 25 | 2.675 |  |
| Other visits | |  |  |  |  |
|  | Number of patients | 101 |  |  |  |
|  | Visit 2 (fitting) | 30 | 176 | 353 |  |
|  | Visit 3 (follow up) | 15 | 176 |  |  |
|  | Visit 4 (follow up) | 15 | 176 |  |  |
|  | Visit 5 (follow up) | 15 | 176 |  |  |
|  | Visit 6 (follow up) | 15 | 176 |  |  |
|  | Visit 7 (follow up) | 15 | 176 |  |  |
|  | Total costs | 101 | 353 | 35.636 |  |
| *Equipment costs* | |  |  |  |  |
|  | Hearing aids | 101 | 1.727 | 174.455 |  |
| Total costs | |  |  |  | **440.975** |
|  |  |  |  |  |  |
| **D. Assessment and follow up of patients presented directly at secondary care level** | | | |  |  |
| *Number of patients* | | 799 |  |  |  |
| *Costs of outpatient visits* | | unit (minutes) | unit cost (15 min) | costs |  |
| Visit 1 (consultation) | |  |  |  |  |
|  | Number of patients | 799 |  |  |  |
|  | Costs of outpatient visits | 30 | 176 | 353 |  |
|  | Total costs | 799 | 353 | 281.909 |  |
| Visit 1 (impression making) | |  |  |  |  |
|  | Number of patients | 130 |  |  |  |
|  | Mould impression materials | 1 | 25 | 25 |  |
|  | Total costs | 130 | 25 | 3.250 |  |
| Other visits | |  |  |  |  |
|  | Visit 2 (fitting) | 30 | 176 | 353 |  |
|  | Visit 3 (follow up) | 15 | 176 | 176 |  |
|  | Visit 4 (follow up) | 15 | 176 | 176 |  |
|  | Visit 5 (follow up) | 15 | 176 | 176 |  |
|  | Visit 6 (follow up) | 15 | 176 | 176 |  |
|  | Visit 7 (follow up) | 15 | 176 | 176 |  |
|  | Total costs | 111 | 1.235 | 137.073 |  |
| *Equipment costs* | |  |  |  |  |
|  | Hearing aids | 111 | 1.727 | 191.728 |  |
| Total costs | |  |  |  | **613.961** |

| Appendix Table 2. Patient volumes and resource use in program 2 | |  |  |  |  |
| --- | --- | --- | --- | --- | --- |
| **Patient numbers** | | Kaniyambadi | Conch | total |  |
|  |  |  |  |  |  |
| At camps | |  |  |  |  |
|  | People screened | 1.188 | 460 | 1.648 |  |
|  | Consultations at 2nd level hospital, referred through camps | 400 | 134 | 534 |  |
|  | Impressions taken | 133 | 32 | 165 |  |
|  | Fitted with hearing aid | 131 | 32 | 163 |  |
| Directly at clinic | |  |  |  |  |
|  | Patients consulted | 202 | NA | 202 |  |
|  | Impressions taken | 40 | NA | 40 |  |
|  | Hearing aid Fitted | 32 | NA | 32 |  |
|  |  |  |  |  |  |
| **A. Fixed project costs** | | Units | Unit price | Costs | **Total costs** |
| *Training costs* | |  |  |  |  |
|  | Per diem for CHW trained (during 3 months, 3300 per month) | 3 | 9.900 | 29.700 |  |
|  | Annualized cost (useful life 5 years) |  |  | 6.485 |  |
| *Personnel* | |  |  |  |  |
|  | Project coordinator (5% FTE) | 1 | 580.598 | 29.030 |  |
|  | Community Hearing Workers (full-time) | 3 | 90.779 | 272.337 |  |
|  | Total costs |  |  | 301.367 |  |
| *Equipment (unit costs are annualized costs)* | |  |  |  |  |
|  | Otoscope | 3 | 293 | 880 |  |
|  | Audiometer | 3 | 5.459 | 16.377 |  |
|  | Ice box | 1 | 152 | 152 |  |
|  | Total costs |  |  | 17.409 |  |
| Total costs | |  |  |  | **325.261** |
|  |  |  |  |  |  |
| **B. Screening in community** | |  |  |  |  |
| *Motivation campaigns* | |  |  |  |  |
|  | Number of motivation campaigns | 27 |  |  |  |
|  | Number of villages covered | 94 |  |  |  |
|  | Travel costs (km traveled) | 1.060 | 7 | 7.420 |  |
|  | Amplifier costs | 27 | 300 | 8.100 |  |
|  | Health extension material costs (all campaigns) |  |  | 650 |  |
|  | Health extension worker | 27 | 250 | 6.750 |  |
|  | Health educator (4 hours per motivation campain) | 27 | 122.000 | 6.863 |  |
|  | Driver (4 hours per motivation campaign) |  | 71.500 | 4.022 |  |
|  | Total costs |  |  | 33.804 |  |
| *Camps* | |  |  |  |  |
|  | Number of camps | 63 |  |  |  |
|  | Number of villages covered | 94 |  |  |  |
|  | Travel costs (km traveled) | 1.681 | 7 | 11.767 |  |
|  | Driver (4 hours per motivation campaign) |  | 71.500 | 18.769 |  |
|  | Total costs |  |  | 30.536 |  |
| Total costs | |  |  |  | **64.340** |
|  |  |  |  |  |  |
| **C. Follow up of screened patients at secondary care level** | | unit (minutes) | unit cost (15 min) | costs |  |
| Visit 1 (consultation) | |  |  |  |  |
|  | Number of patients | 534 |  |  |  |
|  | Costs of outpatient visits | 31 | 176 | 365 |  |
|  | Total costs | 534 | 365 | 194.624 |  |
| Visit 1 (impression making) | |  |  |  |  |
|  | Number of patients | 165 |  |  |  |
|  | Mould impression materials | 1 | 25 | 25 |  |
|  | Total costs | 165 | 25 | 4.137 |  |
| Other visits | |  |  |  |  |
|  | Number of patients | 163 |  |  |  |
|  | Visit 2 (fitting) | 30 | 176 | 353 |  |
|  | Visit 3 (follow up) | 15 | 176 | 176 |  |
|  | Visit 4 (follow up) | 15 | 176 | 176 |  |
|  | Visit 5 (follow up) | 15 | 176 | 176 |  |
|  | Visit 6 (follow up) | 15 | 176 | 176 |  |
|  | Visit 7 (follow up) | 15 | 176 | 176 |  |
|  | Total costs | 163 | 1.235 | 201.288 |  |
| *Equipment costs* | |  |  |  |  |
|  | Hearing aids | 163 | 1.727 | 281.547 |  |
| Total costs | |  |  |  | **681.595** |
|  |  |  |  |  |  |
| **D. Assessment and follow up of patients presented directly at secondary care level** | | | | |  |
| *Number of patients* | | 202 |  |  |  |
| *Costs of outpatient visits* | | unit (minutes) | unit cost (15 min) | costs |  |
| Visit 1 (consultation) | |  |  |  |  |
|  | Number of patients | 202 |  |  |  |
|  | Costs of outpatient visits | 60 | 176 | 706 |  |
|  | Total costs | 202 | 706 | 142.465 |  |
| Visit 1 (impression making) | |  |  |  |  |
|  | Number of patients | 40 |  |  |  |
|  | Mould impression materials | 1 | 25 | 25 |  |
|  | Total costs | 40 | 25 | 989 |  |
| Other visits | |  |  |  |  |
|  | Visit 2 (fitting) | 30 | 176 | 353 |  |
|  | Visit 3 (follow up) | 15 | 176 | 176 |  |
|  | Visit 4 (follow up) | 15 | 176 | 176 |  |
|  | Visit 5 (follow up) | 15 | 176 | 176 |  |
|  | Visit 6 (follow up) | 15 | 176 | 176 |  |
|  | Visit 7 (follow up) | 15 | 176 | 176 |  |
|  | Total costs | 40 | 1.235 | 48.857 |  |
| *Equipment costs* | |  |  |  |  |
|  | Hearing aids | 32 | 1.727 | 55.273 |  |
| Total costs | |  |  |  | **247.584** |

| Appendix Table 3. Patient volumes and resource use in program 3 | |  |  |  |  |
| --- | --- | --- | --- | --- | --- |
| **Patient numbers** | |  |  |  |  |
| Patients | |  |  | 100 |  |
|  |  |  |  |  |  |
| **A. First visit (consultation with audiologist)** | | Units | Unit price | Costs | **Total costs** |
| *Personnel* | |  |  |  |  |
|  | Audiologist (consultation, hearing assessment) (h) | 1 | 142.181 | 68 |  |
| *Equipment (unit costs are annualized costs)* | |  |  |  |  |
|  | Otoscope | 1 | 0 | 0 |  |
|  | Audiometer (per patiënt screened) | 1 | 8 | 8 |  |
| Total costs | |  |  |  | **76** |
|  |  |  |  |  |  |
| **B. First visit (making moud impression)** | |  |  |  |  |
| *Personnel* | |  |  |  |  |
|  | Ear mould technician (h) | 0 | 90.779 | 16 |  |
| *Equipment (unit costs are annualized costs)* | |  |  |  |  |
|  | Mould impression materials | 1 | 25 | 25 |  |
| Total costs | |  |  |  | **41** |
|  |  |  |  |  |  |
| **C. Second visit (collect moulds, hearing aid fitting and counseling)** | | |  |  |  |
| *Personnel* | |  |  |  |  |
|  | Audiologist (hearing aid analysis) (h) | 0 | 142.181 | 36 |  |
|  | Ear mould technician (making ear mould plus adaptation) (h) | 1 | 90.779 | 59 |  |
| *Equipment (unit costs are annualized costs)* | |  |  |  |  |
|  | Cost per hearing aid (purchase costs)* | 1,5 | 1.680 | 2.520 |  |
|  | Real ear analyzer (annualized costs) | 1 | 88 | 88 |  |
| Materials | |  |  |  |  |
|  | Mould materials (per hearing aid) | 1 | 15 | 15 |  |
| Total costs | |  |  |  | **2.718** |
|  |  |  |  |  |  |
| **D. Follow up visits (five in total)** | |  |  |  |  |
| *Personnel costs* | |  |  |  |  |
|  | Audiologist (hearing aid analysis) (h)(five visits) | 1 | 142.181 | 93 |  |
|  | Costs of all fllow-up visits per patient |  |  |  |  |
| Total costs | |  |  |  | **93** |
|  |  |  |  |  |  |
| **E. Costs of outpatient visits (units in minutes)** | |  |  |  |  |
|  | Visit 1 | 55 | 261 | 957 |  |
|  | Visit 2 | 29 | 261 | 505 |  |
|  | Visit 3 (follow up) | 15 | 261 | 261 |  |
|  | Visit 4 (follow up) | 15 | 261 | 261 |  |
|  | Visit 5 (follow up) | 15 | 261 | 261 |  |
|  | Visit 6 (follow up) | 15 | 261 | 261 |  |
|  | Visit 7 (follow up) | 15 | 261 | 261 |  |
| Total costs | |  |  |  | **2.766** |
|  |  |  |  |  |  |
| **Total program** | |  |  |  |  |
|  | Total costs (A+B+C+D) |  |  |  | **569.332** |
|  | Total costs per hearing aid fitted |  |  |  | **5.693** |

‘* On average. 50 people received monaural hearing aids, and 50 people received binaural hearing aids

| **Appendix Table 4.** Household treatment patterns and costs prior and in present programs | | | | | | | |
| --- | --- | --- | --- | --- | --- | --- | --- |
|  |  |  | Program 2 |  | Program 3 |  | Program 1 |
| **Employment** | | |  |  |  |  |  |
|  | Main activities of treated individuals (n) | |  |  |  |  |  |
|  |  | Own farm activities | 24 |  | 3 |  | 16 |
|  |  | Casual labour | 27 |  | 5 |  | 21 |
|  |  | Long term agricultural employment | 6 |  | 2 |  | 7 |
|  |  | Salaried employment | 18 |  | 7 |  | 23 |
|  |  | Petty business / trade / manufacturing | 10 |  | 7 |  | 18 |
|  |  | Other | 10 |  | 8 |  | 19 |
|  |  | Total | 95 |  | 32 |  | 104 |
| **Treatment pattern and household costs prior to present program** | | |  |  |  |  |  |
|  | Months suffered from hearing impairment prior to enrolment in present program | | 116 |  | 127 |  | 139 |
|  | Individual consulted any provider before enrolment in present program | |  |  |  |  |  |
|  |  | Yes | 27 |  | 1 |  | 24 |
|  |  | No | 22 |  | 9 |  | 13 |
|  |  | Total | 49 |  | 10 |  | 37 |
|  | Site of first consultation ever for treatment of hearing disorders (n) | |  |  |  |  |  |
|  |  | Indiginous practitioner | 1 |  | 0 |  | 1 |
|  |  | Village health worker | 1 |  | 0 |  | 1 |
|  |  | Public hospital | 8 |  | 1 |  | 6 |
|  |  | Private provider | 16 |  | 0 |  | 15 |
|  |  | Other | 1 |  | 0 |  | 1 |
|  |  | Total | 27 |  | 1 |  | 24 |
|  | Costs related to first consultations prior to present program (Rs) | |  |  |  |  |  |
|  |  | Costs of fees, drugs, tests etc | 531 |  | 2 |  | 253 |
|  |  | Costs of transport to health provider of person and family | 65 |  | 100 |  | 105 |
|  |  | Income loss because of seeking and undergoing care of person and family | 36 |  |  |  | 90 |
|  | Number of people having second or third consultations (n) | | 7 |  | 4 |  | 13 |
| **Treatment pattern and household costs related to present program** | | |  |  |  |  |  |
|  | Site of first contact in present program (n) | |  |  |  |  |  |
|  |  | Hearing camp | 42 |  | 0 |  | 15 |
|  |  | Health facility | 4 |  | 10 |  | 19 |
|  |  | Other | 3 |  | 0 |  | 3 |
|  |  | Total | 49 |  | 10 |  | 37 |
|  | Costs related to first contact in present program (Rs) | |  |  |  |  |  |
|  |  | Costs of fees, drugs, tests etc | 1 |  | 107 |  | 8 |
|  |  | Number of accompanying persons | 0,3 |  | 0,6 |  | 0,6 |
|  |  | Costs of food and transport of seeking and undergoing care of person and family | 2 |  | 39 |  | 25 |
|  |  | Number of days absent from work | 0,3 |  | 0,9 |  | 0,7 |
|  |  | Income loss because of seeking and undergoing care of person and family | 17 |  | 37 |  | 53 |
|  | Site of follow-up consultation in present program (n) | |  |  |  |  |  |
|  |  | Hearing camp | 0 |  | 0 |  | 1 |
|  |  | Health facility | 48 |  | 10 |  | 36 |
|  |  | Other | 1 |  | 0 |  | 0 |
|  |  | Total | 49 |  | 10 |  | 37 |
|  | Costs related to follow-up consultation in present program (Rs) | |  |  |  |  |  |
|  |  | Costs of fees, drugs, tests etc | 0 |  | 0 |  | 0 |
|  |  | Number of accompanying persons | 0,2 |  | 0,2 |  | 0,4 |
|  |  | Costs of food and transport of seeking and undergoing care of person and family | 10 |  | 25 |  | 24 |
|  |  | Number of days absent from work | 0,4 |  | 0,6 |  | 0,6 |
|  |  | Income loss because of seeking and undergoing care of person and family | 12 |  | 17 |  | 40 |
