# Supplementary material for: Costs and health effects of screening and delivery of hearing aids in Tamil Nadu, India: an observational study
Source: BMC Public Health. 2009 May 12;9:135. doi: 10.1186/1471-2458-9-135 (PMC2695455; doi:10.1186/1471-2458-9-135)
Supplement: Additional file 2 — Appendix questionnaire. Questionnaire about the costs of hearing problems and its treatment. [file 1471-2458-9-135-S2.doc]

**Appendix questionnaire**

**COSTS OF HEARING PROBLEMS AND ITS TREATMENT**

**Questionnaire to be administered at 1^st^ follow-up visit**

Patient identification code: l__________l

**INTRODUCTION**

This questionnaire consists of three parts. In part A, I will ask you some general questions about your income and on the effect of the hearing problem on your income. In part B, I ask you questions about the health providers you contacted for treating your hearing problems before coming to this health facility. In part C I will ask you questions on costs incurred while you are treated for your hearing problem.

**PART A**

I start with the questions on your income and the effects of the hearing problem on your income.

|  |  | Note for interviewer |  |
| --- | --- | --- | --- |
| 1 | How many months have you suffered from your hearing problems before you were helped? | Report number of months. If zero, write 0. |  |
| 2 | What was your main activity before you had your hearing problems? | See table below, and report number of activity. |  |
| 3 | What was your monthly income of these activities? | Record amount in Rupees. If zero, write 0. |  |
| 4 | Have the activities changed because of your hearing problems? | Yes 1 (continue)  No 2 (go to part B) |  |
| 5 | What are your main activities now? | See table below, and report number of activity. |  |
| 6 | What is your monthly income now? | Record amount in Rupees. If zero, write 0. |  |

| **Activity** | |
| --- | --- |
| Own farm activities 1  Casual labour (farm and non-farm) 2  Long-term agri. employee 3  Salaried employment 4  Personal (Jajmani) services 5  Petty business/ trade/ manufacturing 6  Major business/ trade/ manufacturing 7  Collection/ foraging 8 | Charity/ alms 9  Unemployed 10  Student 11  Domestic duties only 12  Retired/ too old 13  Disabled/ handicapped 14  Not working 15  Other (specify) 16 |

**PART B**

I now ask you questions about the health providers you contacted for treating your hearing problems before coming to this health facility.

| 7 | Have you consulted anyone for treating your hearing problem prior to your consultation here? For example a doctor, nurse, traditional healer, village health worker, pharmacist or other practitioner? | Yes 1 (continue)  No 2 (go to part C) |  |
| --- | --- | --- | --- |

I will now read several places where you might have been treated or purchased items needed to treat your hearing problem

Indigenous practitioner.......................... . 1

Faith healer/ religious person............ . 2

Chemist (e.g. pharmacy)........................... 3

Village health worker/nurse practitioner.... 4

Compounder (i.e. quack)............ ... .5

Govt. doctor, PHC, CHC, sub-centre........ .6

Govt. doctor, hospital................................ .7

Govt. doctor, elsewhere............................ .8

Private doctor............................................ .9

Charitable/ NGO doctor ......... 10

Other (specify)......................................... 11

| 8 | Of the places you visited, which was the **first** one where you sought care? | Record number of place |  |
| --- | --- | --- | --- |
| 9 | At this place, how much money did you spend on registration fees, drugs, tests etc? | Record the total amount in Rupees. If zero, write 0 |  |
| 10 | To travel to this place, how much money did you and any accompanying persons spend on transportation and food costs? | Record the total amount in Rupees. If zero, write 0 |  |
| 11 | How much income have you and any accompanying persons lost because of your travel to this place? | Record the total amount in Rupees. If zero, write 0 |  |
| 12 | Of the places you visited, which was the **second** one where you sought care? | Record number of place |  |
| 13 | At this place, how much money did you spend on registration fees, drugs, tests etc? | Record the total amount in Rupees. If zero, write 0 |  |
| 14 | To travel to this place, how much money did you and any accompanying persons spend on transportation and food costs? | Record the total amount in Rupees. If zero, write 0 |  |
| 15 | How much income have you and any accompanying persons lost because of your travel to this place? | Record the total amount in Rupees. If zero, write 0 |  |
| 16 | Of the places you visited, which was the **third** one where you sought care? | Record number of place |  |
| 17 | At this place, how much money did you spend on registration fees, drugs, tests etc? | Record the total amount in Rupees. If zero, write 0 |  |
| 18 | To travel to this place, how much money did you and any accompanying persons spend on transportation and food costs? | Record the total amount in Rupees. If zero, write 0 |  |
| 19 | How much income have you and any accompanying persons lost because of your travel to this place? | Record the total amount in Rupees. If zero, write 0 |  |

**Part C**

Now I will ask you questions on costs incurred for the current treatment of your hearing problem.

| 20 | How many visits did you make so far to the camp and/or health facility for your hearing problems, excluding this visit? | Record the total number of visits. If zero, write 0 |  |
| --- | --- | --- | --- |
| 21 | Where did you get your **first** consultation for your hearing problems? | Hearing camp....1  Health facility.....2  Other.................3 |  |
| 22 | At this place, how much money did you spend on registration fees, drugs, tests etc? | Record the total amount in Rupees. If zero, write 0 |  |
| 23 | Did any relatives and/or friends come with you? If yes, how many? | Record the total number of persons. If zero, write 0 |  |
| 24 | At this place, how much money did you and your accompanying persons spend on food, lodging costs etc? | Record the total amount in Rupees. If zero, write 0 |  |
| 25 | To travel to this place, how much money did you and your accompanying persons spend on transport costs? | Record the total amount in Rupees. If zero, write 0 |  |
| 26 | How many days were you absent from work because of traveling and undergoing care at this place? | Record the total number of days. If zero, write 0 |  |
| 27 | How much income have you lost because of your travel to this place? | Record amount in Rupees. If zero, write 0. |  |
| 28 | How much income have any accompanying persons lost because of travel to this place? | Record the total amount in Rupees. If zero, write 0 |  |
| 29 | Where did you get your **second** consultation for your hearing problems? | Village...............1  Health facility.....2  Other.................3 |  |
| 30 | At this place, how much money did you spend on registration fees, drugs, tests etc? | Record the total amount in Rupees. If zero, write 0 |  |
| 31 | Did any relatives and/or friends come with you? If yes, how many? | Record the total number of persons. If zero, write 0 |  |
| 32 | At this place, how much money did you and your accompanying persons spend on food, lodging costs etc? | Record the total amount in Rupees. If zero, write 0 |  |
| 33 | To travel to this place, how much money did you and your accompanying persons spend on transport costs? | Record the total amount in Rupees. If zero, write 0 |  |
| 34 | How many days were you absent from work because of traveling and undergoing care at this place? | Record the total number of days. If zero, write 0 |  |
| 35 | How much income have you lost because of your travel to this place? | Record amount in Rupees. If zero, write 0. |  |
| 36 | How much income have any accompanying persons lost because of travel to this place? | Record the total amount in Rupees. If zero, write 0 |  |
| 37 | How much income have these accompanying persons lost because of traveling to this place? | Record the total amount in Rupees. If zero, write 0 |  |
| 38 | Where did you get your **third** consultation for your hearing problems? | Village...............1  Health facility.....2  Other.................3 |  |
| 39 | At this place, how much money did you spend on registration fees, drugs, tests etc? | Record the total amount in Rupees. If zero, write 0 |  |
| 40 | Did any relatives and/or friends come with you? If yes, how many? | Record the total number of persons. If zero, write 0 |  |
| 41 | At this place, how much money did you and your accompanying persons spend on food, lodging costs etc? | Record the total amount in Rupees. If zero, write 0 |  |
| 42 | To travel to this place, how much money did you and your accompanying persons spend on transport costs? | Record the total amount in Rupees. If zero, write 0 |  |
| 43 | How many days were you absent from work because of traveling and undergoing care at this place? | Record the total number of days. If zero, write 0 |  |
| 44 | How much income have you lost because of your travel to this place? | Record amount in Rupees. If zero, write 0. |  |
| 45 | How much income have any accompanying persons lost because of travel to this place? | Record the total amount in Rupees. If zero, write 0 |  |
| 46 | How much income have these accompanying persons lost because of traveling to this place? | Record the total amount in Rupees. If zero, write 0 |  |
| 47 | Now we ask you questions about the **current** consultation for your hearing problems [interviewer, please fill in place of current consultation]. | Village...............1  Health facility.....2  Other.................3 |  |
| 48 | At this place, how much money did you spend on registration fees, drugs, tests etc? | Record the total amount in Rupees. If zero, write 0 |  |
| 49 | Did any relatives and/or friends come with you? If yes, how many? | Record the total number of persons. If zero, write 0 |  |
| 50 | At this place, how much money did you and your accompanying persons spend on food, lodging costs etc? | Record the total amount in Rupees. If zero, write 0 |  |
| 51 | To travel to this place, how much money did you and your accompanying persons spend on transport costs? | Record the total amount in Rupees. If zero, write 0 |  |
| 52 | How many days were you absent from work because of traveling and undergoing care at this place? | Record the total number of days. If zero, write 0 |  |
| 53 | How much income have you lost because of your travel to this place? | Record amount in Rupees. If zero, write 0. |  |
| 54 | How much income have any accompanying persons lost because of travel to this place? | Record the total amount in Rupees. If zero, write 0 |  |
| 55 | How much income have these accompanying persons lost because of traveling to this place? | Record the total amount in Rupees. If zero, write 0 |  |
